# International Efforts and Next Steps to Advance COVID-19 Vaccines Research and Production in Low- and Middle-Income Countries

**DOI:** 10.3390/vaccines10010042

**Published:** 2021-12-29

**Authors:** Li Du, Meng Wang, Vera Lúcia Raposo

**Affiliations:** 1 Faculty of Law, University of Macau, Avenida da Universidade, Taipa 999078, Macao; mirandawm@yeah.net; 2 Faculty of Law, Coimbra University, 3004-531 Coimbra, Portugal; vera@fd.uc.pt

**Keywords:** LMICs, COVID-19 vaccines, global health law, vaccine distribution, vaccine equity, global vaccine certificate

## Abstract

Equitable and efficient distribution of COVID-19 vaccines continues to be a key issue in global health, and a targeted approach is needed to meet the World Health Organization’s world vaccination targets. Although some low- and middle-income countries (LMICs) are developing their own vaccines to address the distribution problem, legal and technical challenges have had a negative impact on productivity. This article explores relevant international legal instruments that can enable faster research and development of COVID-19 vaccines in LMICs, focusing on the role of biosafety standards, biological materials transfer, and key knowledge sharing. Our analysis has established that the potential of existing global health legal instruments has yet to be realized in order to close the productivity gap in LMICs and strengthen their vaccine manufacturing capacity. Additionally, mutual recognition of vaccine efficacy has become a new challenge for achieving global vaccination targets. We argue that the World Health Organization should continue its leading position by developing a more practical and targeted framework to help LMICs overcome challenges arising from technology transfer, knowledge sharing, and politics.

## 1. Introduction

Equitable and fair distribution of vaccines around the world is the key to controlling the spread of COVID-19 infections. The COVID-19 Vaccines Global Access (COVAX) scheme was developed to achieve this goal, raising public hopes for expanded access. Under ideal conditions, vaccine doses would be distributed proportionally to participating countries to minimize wealth disparities in global health. While these expectations are reasonable, their implementation has turned out difficult in practice. As of November 26, 2021, the COVAX has shipped just 537 million doses [1], well below its current goal of distributing 1.425 billion doses by the end of 2021 [2].

Vaccine nationalism has dramatically impacted the function of COVAX [3]. Countries with vaccine production capacity inevitably prioritize their national supply, and so do rich countries that can secure advanced purchases from vaccine manufacturers. One example is the outbreak in India, which led to a temporary suspension of COVID-19 vaccine exports and negatively affected the scheme [4]. Many nations, particularly low- and middle-income countries (LMICs), did not receive vaccine deliveries as scheduled and had to delay vaccination efforts. Some countries such as China and Russia export their own manufactured vaccines to LMICs, who suffer from insufficient vaccination rates. For instance, China has consistently provided vaccine assistance to countries such as Laos, Jamaica, and Peru, and the Russian-produced Sputnik V vaccine has been ordered by countries such as Vietnam and Argentina [5]. Member states of the European Union have also donated millions of COVID-19 vaccines to lower-income economies around the world, including Nigeria, Syria, and Ghana, among others [6]. However, the problem of vaccines shortage in LMICs has not been resolved. As a recent study indicates, COVID-19 vaccination in the Global South has lagged, with less than 10% vaccinated people in LMICs compared to more than 60% in high-income countries [7]. The existing ineffective and inequitable distribution mechanism, combined with the continuously evolving SARS-CoV-2 variants, requires swift action to improve access to vaccines in LMICs. If the status quo remains unchanged, as indicated by Taylor, low-income countries will only reach mass vaccination goals in 2023 and, potentially, herd immunity around that time or even later [8]. The approval of booster shots will further worsen the supply to LMICs, according to World Health Organization (WHO) [9].

Confronted with limited vaccine supplies and urgent needs for mass vaccination, some LMICs have started to develop COVID-19 vaccines locally rather than waiting for the promised vaccine doses. For example, Iran and Cuba have engaged in vaccine research and development initiatives [10,11]. Their vaccine development efforts seem promising. For instance, the COVID-19 vaccine developed by Cuba (i.e., the Abdala vaccine) was authorized by the Cuban regulatory agency for adult injection. Although this vaccine has not yet been approved by the WHO, it has been ordered by countries like Venezuela and Vietnam [12].

The manufacturing of vaccines by these LMICs provides hope for increased access to vaccination in the region. However, reliance on independent national vaccine development strategies is not without caveats and may further complicate the technological and societal challenges of the post-pandemic era. Previous studies on vaccine production in LMICs indicate that biosafety standards, as well as requirements for the transfer of biological materials and key knowledge sharing, are the main challenges when manufacturing vaccines. Here, we critically analyze the applicable international law and international guiding principles that enable vaccine production in LMICs [13,14], including those recent instruments and special arrangement documents that allow addressing legal and technical issues in the production of COVID-19 vaccines. We argue that, notwithstanding the international community’s current failure to distribute COVID-19 vaccines equitably, international efforts need to continue to play a key role in regulating and promoting vaccine research and production in LMICs.

## 2. Legal Challenges to Vaccine Development in LMICs Prior to the COVID-19 Pandemic

### 2.1. Biosafety Standards

Biosafety is of utmost importance in developing and producing safe and efficient vaccines, including vaccines for SARS-CoV-2 in LMICs. At the international level, organizations such as WHO, the International Council for Harmonization of Technical Requirements for Pharmaceuticals for Human Use, and the International Organization for Standardization have established guidelines and standards to ensure laboratory biosafety and risk management in manufacturing biological products. For instance, Good Manufacturing Practices (GMP), which include biosafety requirements for drugs and medical products, are required by WHO and other United Nations (UN) agencies conducting prequalification for vaccine procurement [15]. However, appropriate standards for quality and biosafety management in vaccine manufacturing are rarely incorporated in the national laws of LMICs. Only 14 developing countries had met the WHO certification standards in vaccine manufacturing by 2017 [16]. The WHO Laboratory Biosafety Manual (4th edition), which was updated in December 2020, outlines best practices and establishes procedures for the assessment and management of biosafety risks. The guidelines, however, are soft law measures that are not legally binding [17]. Each member state is only encouraged to take them as a reference for establishing their own domestic rules. Consequently, inconsistencies and gaps in the laboratory biosafety and risk control for vaccine manufacturing continue to exist and have been exacerbated due to the increased scale of COVID-19 vaccine production in LMICs.

### 2.2. Transfer of Biological Materials and Knowledge Sharing

A global approach to knowledge sharing and transfer of biomaterials is required for success in vaccine research and development in LMICs [18]. Key knowledge, in this case, comprises patented technologies, as well as skills and know-how relevant to vaccine production. Biological materials include human specimens, isolated viruses with human pandemic potentials, and candidate vaccine viruses for specific diseases. However, there are still limited options for LMICs to gain access to required vaccine technology and intellectual property. Presently, the Pandemic Influenza Preparedness Framework for the Sharing of Influenza Viruses and Access to Vaccines and Other Benefits (PIP Framework) issued by the WHO in 2011 is the only international legal means that provides specific measures for biological materials transferring and knowledge sharing for vaccine research and production; however, it is mostly applicable for research on H5N1 influenza. Under the PIP Framework, WHO proposed the Standard Material Transfer Agreements (SMTAs) for influenza vaccine laboratories and manufacturers. The SMTAs set out the conditions for biological materials transfer from one party to another and sharing intellectual property rights (IPRs) applications and licenses. For example, both parties are required to ensure the handling of biological materials is consistent with the WHO guidelines and national biosafety regulations.

Even with the WHO’s influenza vaccine technology transfer plans under the PIP Framework, vaccine knowledge sharing and influenza vaccine manufacturing capacity in LMICs have not improved dramatically. The number of facilities that can produce influenza vaccines is still limited after more than 10 years of development [19]. There is clearly a discrepancy between the vision of supporting vaccines as international public goods and the reality of open access to patented technology and relevant knowledge. If no effective measures are taken quickly, the current challenges in COVID-19 vaccine research and development in LMICs will continue to persist.

Important reasons for the existing vaccine development gap are the low manufacturing standards and poor infrastructure in LMICs. If laboratories do not meet biosafety and other technical standards, such as the SMTAs proposed in PIP Framework, LMICs would not be able to receive biological materials transferred from providing partners. Sample sharing in the Ebola epidemic is a good example of this issue. Due to biosafety concerns about handling highly infectious Ebola samples, scientists in Sierra Leone, Guinea, and Liberia were unable to access the biomaterials required for their research [20]. Access to biological materials and key knowledge, however, is not sufficient as many LMICs experience serious infrastructure challenges that diminish their production capacity, and their vaccine production often does not meet GMP requirements.

## 3. International Efforts during the COVID-19 Pandemics

Since May of 2020, the international community has consistently devoted efforts to helping LMICs address the challenges and improve domestic capacities for manufacturing vaccines for COVID-19 (Table 1). For example, the UN system, including the UN Conference on Trade and Development, and international intergovernmental organizations, such as the WHO and the Organization for Economic Cooperation and Development (OECD), have developed policy recommendations and initiatives to support LMICs in establishing GMP-compliant plants and facilitating knowledge-sharing about COVID-19 vaccines. Under the World Trade Organization (WTO) system, a proposal for IPRs exemptions for COVID-19 vaccines was put forward. Several patent sharing platforms, e.g., the WHO COVID-19 Technology Access Pool (C-TAP) and its partner VaxPaL, have also been launched for vaccine developers to voluntarily share COVID-19-related patents, knowledge, and data. In July 2021, WHO spearheaded a technology transfer hub in South Africa to assist African LMICs in acquiring know-how and production licenses about the mRNA vaccines. The organization has been negotiating and coordinating with vaccine manufacturers such as Pfizer and Moderna to transfer technology or share knowledge through this hub [21]. On 16 November 2021, Pfizer announced that it would grant the Medicines Patent Pool (MPP) a royalty-free license for its COVID-19 antiviral pills so that manufacturers in LMICs have access to its formula [22].

The above-mentioned international instruments and arrangements reflect the efforts of the international community to narrow the vaccine production gap between developed and developing countries. For instance, by establishing regional vaccine technology transfer centers as mechanisms for patent sharing and expanding licensing agreement, the WHO, in collaboration with vaccine exporting countries and pharmaceutical companies, have developed resolutions to help LMICs overcome their technology and knowledge gaps, as well as legal barriers. As stated in the Strategy to Achieve Global COVID-19 Vaccination by mid-2022, it is estimated that more agreements will be reached in the international community in coming years, which would potentially increase vaccine production in LMICs.

Regardless, only limited improvement has been made under the new guidelines to increase capabilities for manufacturing COVID-19 vaccines in LMICs. Further sustained efforts are needed to build biosafety-compliant infrastructures that can receive vaccine technology transfer and develop appropriate domestic regulations on biosafety risks management and control. In addition, little progress has been made in terms of gaining IPRs and transferring key knowledge under existing regimes. One example is Bolivia, which filed a request for a vaccine patent waiver to access Johnson & Johnson’s vaccine under the compulsory licenses process within the Agreement on Trade-Related Aspects of Intellectual Property Rights (TRIPS) in May 2021. However, until recently, Biolyse Pharma, which manufactures vaccines in Bolivia, has not received the requested intellectual property and technology sharing [23]. As a result, Biolyse cannot produce the needed COVID-19 vaccine supplies, even though it has the capacity to produce 50 million doses per year [24]. Moreover, most vaccine manufacturers in these countries lack high-level technical expertise, which presents further challenges for gaining access to best practices and know-how. A recent study has shown that the number of LMICs that can currently translate shared key knowledge into production capacity is still a minority [25].

In addition, new challenges for COVID-19 vaccine production in LMICs have emerged. For instance, there are often disagreements between authorities at the national and international levels regarding the recognition of vaccines produced by different companies or countries. Although nine vaccines have been included on the WHO Emergency Use List (EUL), countries have the discretion to use their own regulatory approval to import and administer COVID-19 vaccines [26]. With the development of new policies for reopening borders, individual states are likely to pursue their own policies about recognizing vaccine certificates and vaccine efficacy when issuing visas for foreign travelers. The discrepancy in domestic standards will inevitably trigger conflicts between countries on efficacy accreditation.

France, for example, had previously granted entry visas only to travelers vaccinated with the four vaccines only approved by the European Medicines Agency (EMA), i.e., Pfizer, Moderna, Janssen, and AstraZeneca. The restriction has just recently been lifted. However, for international travelers who have already received two shots of other vaccines from the WHO EUL, the French Ministry of Foreign Affairs still requires an additional booster (Moderna or Pfizer) [27]. This exclusion from the recognition list constitutes discrimination against foreign travelers inoculated with other vaccines and limits their mobility. If the current policy variations between countries continue, vaccines developed by LMICs will likely face similar challenges. This can have a further impact on vaccine uptake, as many citizens of those countries may become hesitant to get vaccinations in their home jurisdictions.

## 4. What’s Next for Vaccine Development in LMICs

Our analysis of the existing international law and other applicable non-binding agreements and policy frameworks indicates that international efforts so far have not been sufficient to substantially help LMICs to increase their vaccine production capacity. A more proactive and practical approach should be developed to address barriers to vaccine production in LMICs. We all, as a global community, should be engaged in sustained efforts for the future, as expressed in goal 3.b of the 2030 Agenda for Sustainable Development.

As stated in the Constitution of the WHO (Article 2), the organization is responsible for improving public health and promoting the establishment of international standards for biological products. Article 44 of the International Health Regulations (2005) also emphasizes the authority of WHO in promoting technical co-operation among member states to improve capacity to respond to public health crises, particularly in developing countries. As supported by relevant laws and policy frameworks shown in Table 2, WHO could play a more proactive role in improving the vaccine production capacity in LMICs for the common good of all humanity in the face of global public health crises. The current mechanisms are mainly based on soft law measures, which are not legally binding to member states, rendering the approach weak in terms of practical solutions and implementation. Moreover, under this scenario, there is a risk that vaccines are misused politically as a token payment for political support from the LMICs [28]. In this regard, we argue that the WHO should take a more proactive role in developing international agreements and policy frameworks with legally binding effects on member states that can substantially improve vaccine manufacturing capabilities in LMICs.

Specifically, the first step towards the safe and efficient manufacturing of COVID-19 vaccines is to achieve a minimum level of biosafety standards in LMICs. The WHO Strategic Advisory Group of Experts on Immunization (SAGE) may undertake an advisory role in assisting LMICs to develop the minimum level of biosafety based on the GMP standards. By collecting and analyzing current biosafety requirements in LMICs with vaccine manufacturing capacities, SAGE could identify gaps between the ability of these countries to control biosafety risks and the current international standards and develop targeted recommendations for improvement. Moreover, similarly to the WHO strategy in the novel oral polio vaccine approval, a WHO-led expert panel with highly trained and experienced GMP auditors could help vaccine manufacturers in LMICs improve their biosafety risk management and obtain WHO EUL for vaccines [29].

Based on the Cartagena Protocol on Biosafety (Article 22), it is advisable for the WHO to establish specialized technical assistance groups to assist LMICs in rapidly building laboratories for COVID-19 vaccine research. The establishment of a biosafety-compliant infrastructure will facilitate the delivery of biological materials for vaccines research and production. A special vaccine knowledge-sharing team dispatched by the WHO can be established to collect, review, and release vaccine-related technical knowledge to the international community [30]. Moreover, the WHO and other international organizations could play proactive roles in negotiations between pharmaceutical companies and LMICs to improve the existing ineffective mechanisms of “voluntary” disclosure of technology and knowledge. The WHO’s recent efforts in South Africa constitute a great beginning. In this scheme, the organization serves as an intermediary, providing oversight and assurance, which would likely prompt many large academic institutions and vaccine companies to share technology and knowledge with the hub [31]. If the scheme works successfully, it could be adapted and considered for use in other LMICs or regions. Pfizer’s positive approach to formulation sharing of its antiviral pill also provides a good example for vaccine-related knowledge sharing. However, the approach to knowledge sharing advocated in this paper does not require a suspension of existing norms, nor does it constitute a violation of international patent law, since current agreements such as TRIPS provide for exceptions to patent protection in its Articles 30 and 31. Our proposal here is that when existing regimes cannot resolve the problem in a timely manner, new schemes should be established to respond to the urgent need for global pandemic control.

Regarding the international recognition of different vaccines’ efficacy, the WHO’s SAGE and WHO Prequalification Project are responsible for COVID-19 vaccine evaluation. It is necessary for LMICs which develop and manufacture COVID-19 vaccines to follow the WHO’s norms and standards to ensure their vaccines meet the qualification requirements in both national and international markets. Member states need to consider introducing new regulations to recognize the vaccines that the SAGE and prequalification process have already approved. Once the WHO qualifies the vaccines, they are supposed to be viewed as having sufficient efficacy and be recognized by all member states as effective vaccines, as asserted in the Joint COVAX Statement on the Equal Recognition of Vaccines released by the WHO in July 2021.

Lastly, the international community should strive to secure additional budgets for LMICs to achieve progress in improving both biosafety standards and data sharing. The Alliance for Health Policy and Systems Research, which is a partnering organization with the WHO, has already increased budgets or solicited funding specifically for projects in LMICs. New funding opportunities for research and development of COVID-19 vaccines in LMICs should be considered by the WHO.

## 5. Conclusions

The COVID-19 pandemic is still spreading globally. The considerable immunization lag due to shortages in the supply of COVID-19 vaccines threatens to turn LMICs into factories for cultivating SARS-CoV-2 variants. The consequences, if this continues, would be catastrophic. Current imbalances in COVID-19 vaccine supply demonstrate the large gap in vaccine production capacity between high-income countries and LMICs. Discrepancies in vaccine production have put LMICs at a disadvantage, and many countries struggle to obtain sufficient vaccine doses. If this problem is not promptly addressed, LMICs may face the dilemma of poor access to vaccines again in the next global pandemic.

It is urgent for the international community to provide substantial assistance with developing and manufacturing COVID-19 vaccines locally. In the past, the WHO has assisted LMICs countries in influenza vaccine production and has also helped local manufacturers to obtain EUL certification. Now it is crucial to apply valuable knowledge accrued from past experiences and take swift actions to strengthen vaccine production in regions where it is most needed. Developing a more practical and targeted legal and policy framework will help LMICs overcome challenges arising from technology transfer, knowledge sharing, and politics in vaccine production and endorsement. It should be noted that the achievement of this goal would not inevitably result in a breach of existing laws, as the current legal framework the TRIPS already provides for that possibility. Efforts from the WHO, however, are not enough to build sufficient COVID-19 vaccine productive capacity in LMICs. Global partnerships with other stakeholders, i.e., international organizations, governments, pharmaceutical companies, and international investors and technology holders, can play a key role in facilitating COVID-19 vaccine research and production in LMICs. In the words of Peter Singer, adviser to the Director-General of the WHO, “Charity is good, but we cannot rely on charity alone” [7].

## Figures and Tables

**Table 1:** Examples of international legal measures, initiatives, frameworks, and special arrangements for improving COVID-19 vaccine production in LMICs.

International Instruments and Special Arrangements	Specific Content	Practical Effects
Ten Actions to Boost Low and Middle-Income Countries’ Productive Capacity for Medicines 27 May 2020 (United Nations Conference on Trade and Development, UNCTAD)	1. Investment in skills development to ensure GMP-compliant production 2. Sharing COVID-19-related technologies to enable affordable mass production	It only serves as a recommendation and facilitator of international soft law; it is not legally enforceable, and no country has responded to it explicitly. The proposal addresses a gap in the international COVID-19 response by boosting productive capacity in LMICs. UNCTAD will intensify collaboration with five partner agencies, i.e., WHO, The Global Fund, UNICEF, UNIDO, and UNAIDS … at the World Investment Forum later this year to mobilize key global players to commit to longer-term productive capacity building. (Retrieved from Ten Actions to Boost Low and Middle-Income Countries’ Productive Capacity for Medicines; available on the official website of the UNCTAD; updated on 27 May 2020)
The COVID-19 Technology Access Pool, C-TAP Proposed in May 2020 (World Health Organization, WHO)	implementing partners include the Medicines Patent Pool, Open COVID Pledge, UN Technology Bank, and Unitaid. Developers of COVID-19 health technologies and holders of related knowledge, intellectual property, and data are invited to “share their intellectual property, knowledge and data, and join the Solidarity Call to Action.”	Thus far, only a small number of countries have endorsed C-TAP, among them none of the countries that host major R&D and production capacity, nor have any of the principal vaccine manufacturers agreed to participate. (Retrieved from OECD Policy Responses to Coronavirus; available on the official website of OECD; updated on 18 March 2021)
Waiver from Certain Provisions of the Agreement on Trade-Related Aspects of Intellectual Property Rights (TRIPS Agreement) for the Prevention, Containment, and Treatment of COVID-1915–16 October 2020 (It was first introduced to the World Trade Organization, TRIPS Council by India and South Africa)	many countries, especially developing countries, may face institutional and legal difficulties when using TRIPS flexibilities, including the special compulsory licensing mechanism provided for in Article 31bis ... Internationally, there is an urgent call for global solidarity, and the unhindered global sharing of technology and know-how in order that rapid responses for the handling of COVID-19 can be put in place on a real time basis. ... we request that the Council for TRIPS recommends, as early as possible, to the General Council a waiver from the implementation, application, and enforcement of Sections 1, 4, 5, and 7 of Part II of the TRIPS Agreement in relation to prevention, containment, or treatment of COVID-19. ...The waiver should continue until widespread vaccination is in place globally, and most of the world’s population has developed immunity; hence, we propose an initial duration of [x] years from the date of the adoption of the waiver.	…Some 40 members engaged in a substantive discussion on a proposal submitted by India and South Africa for a temporary waiver of certain TRIPS obligations they said would facilitate an appropriate response to COVID-19 …A number of developing and developed country members opposed the waiver proposal, noting that there is no indication that intellectual property rights have been a genuine barrier to accessing COVID-19 related medicines and technologies …Given this range of positions, the Council chair…said that the item would remain suspended as members continue to consider the proposal. Requests for waivers concerning WTO agreements must be submitted initially to the relevant council for consideration. …Unable to complete the consideration of the revised waiver request, the TRIPS Council will therefore continue discussions. (Retrieved from Members discuss intellectual property response to the COVID-19 pandemic, and TRIPS Council agrees to continue discussions on IP response to COVID-19; available on the official website of WTO; updated in October 2020 and July 2021)
Coronavirus (COVID-19) Vaccines for Developing Countries: An Equal Shot at Recovery 4 February 2021 (The Organization for Economic Co-operation and Development, OECD)	... Research and development of vaccines, as well as production capacity, is concentrated in just a few countries in the world, thereby requiring most low- and middle-income countries to import vaccines. A global consensus on principles for equitable access can usefully build on Gavi’s work to establish principles for sharing vaccine doses between countries through COVAX. It should also translate into allocation sequences and distribution mechanisms that are legally binding and can be enforced. Where possible, the transfer of technical know-how to manufacturers in developing countries should be encouraged.	Number of Members: 38 The policy responses from the OECD…providing guidance on the short-term measures needed in affected sectors and … analysis on the longer-term consequences and impacts, paving the way to recovery with coordinated policy responses across countries. However, these responses are only advisory and not actually binding. (Retrieved from Key policy responses from the OECD; available on the official website of OECD; updated in October 2020)
Access to COVID-19 Vaccines: Global Approaches in a Global Crisis 18 March 2021 (The Organization for Economic Co-operation and Development, OECD)	boosting production capacity and increasing supply… also includes facilitating the sharing of intellectual property and knowledge transfer so that supply can be increased in countries in addition to those where production is currently taking place… This includes expanding licensing arrangements to accelerate production of vaccines, as well as coordinated approaches to sharing intellectual property and technology transfer, for example, through participation in the C-TAP or through multilateral approaches in the World Trade Organization.	
VaxPaL Patent and licensing status: last updated on 11 May 2021(The Medicines Patent Pool)	VaxPaL is the Medicines Patent Pool (MPP)’s new patents database devoted to COVID-19 vaccines. MPP is a United Nations-backed public health organization working to increase access to, and facilitate the development of, life-saving medicines for low- and middle-income countries. Through its innovative business model, MPP partners with civil society, governments, international organizations, industry, patient groups, and other stakeholders, …and pool intellectual property to encourage generic manufacture and the development of new formulations. In 2020, MPP’s mandate was temporarily expanded to include COVID-19 treatments. In 2021, it was expanded further to include the licensing of technology with an initial focus on COVID-19 vaccines and pandemic preparedness.	VaxPaL does not accept any legal responsibility for the accuracy of data. The platform does not guarantee it is complete, up to date, or fit for specific purposes. (Retrieved from VaxPaL-COVID-19 vaccines patent landscape; available on the official website of the Medicines Patent Pool; updated in October 2021)
The mRNA vaccine Technology Transfer Hub Formally established in July 2021 (World Health Organization, Afrigen Biologics (PTY) Limited, the Biologicals and Vaccines Institute of Southern Africa (Biovac), the South African Medical Research Council, Africa Centers for Disease Control and Prevention, and the MPP)	The mRNA vaccine Technology Transfer Hub was established in response to the flagrant inequities in access to COVID-19 vaccines in low- and middle-income countries, especially in Africa... the purpose of the hub is to increase access to mRNA vaccines made closer to home by establishing manufacturing capacity using a technology transfer hub model to ensure sustainable vaccine security in future pandemics... The Hub will be an mRNA training facility where the technology is established at industrial scale and clinical development is performed. Interested manufacturers from low- and middle-income countries can receive training and any necessary licenses to the technology. WHO and partners will bring in the production know-how, quality control, and necessary licenses to a single entity to facilitate a broad and rapid technology transfer to multiple recipients around the world.	Plans for the Technology Transfer Hub: 2021–2022: (1) mRNA vaccine technology selection; (2) design and prepare GMP sites in South Africa. 2022–2023: Efficacy studies; Tech Transfer to first spoke site; Clinical trials. 2024: mRNA vaccine approval and commercialization begin. 2025 and beyond: expand mRNA vaccine capabilities of existing manufacturers in LMICs and establish sustainable vaccine capacity in regions where it is limited. (Retrieved from Timeline over the next five years; available on the official website of the Medicines Patent Pool; updated in October 2021)
WHO, UN set out steps to meet world COVID vaccination targets 7 October 2021 (World Health Organization)	Vaccine-producing countries must: allow the free cross-border flow of finished vaccines and raw materials; enable diversified vaccine production, both geographically and technologically, including through non-exclusive and transparent licensing and sharing of know-how to allow transfer of technology and scale-up of manufacturing. COVID-19 vaccine manufacturers must: commit to share know-how more rapidly, facilitate technology transfer, and provide transparent non-exclusive voluntary licenses to ensure that future vaccine supply is reliable, affordable, available, and deployed to every country in volumes and timing that achieves equitable access.	There are no documents or actions that specifically respond to this initiative. However, the international community has shown an open attitude toward sharing knowledge about the new coronavirus epidemic. As an example, U.S. pharmaceutical giant Pfizer has agreed to a license-sharing deal that would allow its experimental COVID-19 drug to be manufactured more widely around the globe. It is an agreement that the company says could give more than half of the world’s population access to the treatment, even as Pfizer rebuffs calls to grant poorer countries access to its coronavirus vaccine formula. (Retrieved from Pfizer to share license for COVID-19 pill, potentially opening up treatment to millions in low-income nations; available on The Washington Post; updated in November 2021)

**Table 2:** Applicable international laws, non-binding measures, and policy frameworks to improve the vaccine production capacity in LMICs.

International Laws and Documents	Specific Content
Constitution of the World Health Organization (It was adopted by the International Health Conference held in New York from 19 June to 22 July 1946, signed on 22 July 1946, and entered into force on 7 April 1948.)	Article 2: In order to achieve its objective, the functions of the Organization shall be: (a) to act as the directing and coordinating authority on international health work; (b) to establish and maintain effective collaboration with the United Nations, specialized agencies, governmental health administrations, professional groups and such other organizations as may be deemed appropriate; ... (g) to stimulate and advance work to eradicate epidemic, endemic and other diseases; ... (i) to promote, in co-operation with other specialized agencies where necessary, the improvement of nutrition, housing, sanitation, recreation, economic or working conditions and other aspects of environmental hygiene; (j) to promote co-operation among scientific and professional groups which contribute to the advancement of health; (k) to propose conventions, agreements and regulations, and make recommendations with respect to international health matters and to perform such duties as may be assigned thereby to the Organization and are consistent with its objective; ... (n) to promote and conduct research in the field of health; (o) to promote improved standards of teaching and training in the health, medical and related professions; (p) to study and report on, in co-operation with other specialized agencies where necessary, administrative and social techniques affecting public health and medical care from preventive and curative points of view, including hospital services and social security; (q) to provide information, counsel and assistance in the field of health; ... (u) to develop, establish and promote international standards with respect to food, biological, pharmaceutical and similar products;
Universal Declaration of Human Rights (It was proclaimed by the United Nations General Assembly in Paris on 10 December 1948.)	Article 1: All human beings are born free and equal in dignity and rights. They are endowed with reason and conscience and should act towards one another in a spirit of brotherhood. Article 22: Everyone, as a member of society, has the right to social security and is entitled to realization, through national effort and international co-operation and in accordance with the organization and resources of each State, of the economic, social, and cultural rights indispensable for his dignity and the free development of his personality.
Charter of the United Nations (It first entered into force on 24 October 1945; the latest amendment was adopted on 20 December 1971 and entered into force on 24 September 1973.)	Article 1: The Purposes of the United Nations are: … in conformity with the principles of justice and international law, adjustment or settlement of international disputes or situations which might lead to a breach of the peace; To develop friendly relations among nations based on respect for the principle of equal rights and self-determination of peoples, and to take other appropriate measures to strengthen universal peace; To achieve international co-operation in solving international problems of an economic, social, cultural, or humanitarian character, and in promoting and encouraging respect for human rights and for fundamental freedoms for all without distinction as to race, sex, language, or religion; and To be a center for harmonizing the actions of nations in the attainment of these common ends. Article 55: With a view to the creation of conditions of stability and well-being which are necessary for peaceful and friendly relations among nations based on respect for the principle of equal rights and self-determination of peoples, the United Nations shall promote: higher standards of living, … and conditions of economic and social progress and development; solutions of international economic, social, health, and related problems; … and universal respect for, and observance of, human rights and fundamental freedoms for all without distinction as to race, sex, language, or religion.
The Cartagena Protocol on Biosafety to the Convention on Biological Diversity (It was adopted on 29 January 2000 and entered into force on 11 September 2003 by the Secretariat of the Convention on Biological Diversity.)	Article 22 Capacity-building: The Parties shall cooperate in the development and/or strengthening of human resources and institutional capacities in biosafety, including biotechnology to the extent that it is required for biosafety, for the purpose of the effective implementation of this Protocol, in developing country Parties… including through existing global, regional, subregional, and national institutions and organizations and, as appropriate, through facilitating private sector involvement. ... in relation to cooperation, the needs of developing country Parties, …for financial resources and access to and transfer of technology and know-how in accordance with the relevant provisions of the Convention, shall be taken fully into account for capacity-building in biosafety. Cooperation in capacity-building shall, subject to the different situation, capabilities, and requirements of each Party, include scientific and technical training in the proper and safe management of biotechnology, and in the use of risk assessment and risk management for biosafety, and the enhancement of technological and institutional capacities in biosafety…
International Health Regulations (2005) (It was originally adopted in 1969 and subsequently amended in 1973 and 1981. The International Health Regulations (2005) were adopted by the 58th World Health Assembly on 23 May 2005 and entered into force on 15 June 2007. The latest revision entered into force for all States Parties on 11 July 2016.)	Article 44 Collaboration and assistance: 2. WHO shall collaborate with States Parties, upon request, to the extent possible, in: (a) the evaluation and assessment of their public health capacities to facilitate the effective implementation of these Regulations; (b) the provision or facilitation of technical cooperation and logistical support to States Parties; and (c) the mobilization of financial resources to support developing countries in building, strengthening and maintaining the capacities provided for in Annex 1. 3. Collaboration under this Article may be implemented through multiple channels, including bilaterally, through regional networks and the WHO regional offices, and through intergovernmental organizations and international bodies.

## Data Availability

No data were analyzed or generated in this study.
